# Matrix Remodeling Enzymes as Potential Fluid Biomarkers of Neurodegeneration in Alzheimer’s Disease

**DOI:** 10.3390/ijms25115703

**Published:** 2024-05-24

**Authors:** Jelena Bašić, Vuk Milošević, Branka Djordjević, Vladana Stojiljković, Milica Živanović, Nikola Stefanović, Aleksandra Aracki Trenkić, Dragan Stojanov, Tatjana Jevtović Stoimenov, Ivana Stojanović

**Affiliations:** 1 Department of Biochemistry, Faculty of Medicine, University of Niš, 18000 Niš, Serbia; brankadjordjevic83@gmail.com (B.D.); vladana93@hotmail.com (V.S.); tjevtovic@yahoo.com (T.J.S.); stojanovicivana38@gmail.com (I.S.); 2 Faculty of Medicine, University of Niš, 18000 Niš, Serbia; vuk.milosevic@gmail.com (V.M.); aaracki@gmail.com (A.A.T.); drstojanov@gmail.com (D.S.); 3 Clinic of Neurology, University Clinical Center Niš, 18000 Niš, Serbia; 4 Center for Radiology, University Clinical Center Niš, 18000 Niš, Serbia; milicazivanovic2601@gmail.com; 5 Department of Pharmacy, Faculty of Medicine, University of Niš, 18000 Niš, Serbia; nikola.z.stefanovic@gmail.com

**Keywords:** Alzheimer’s disease, APOE ε4, hippocampal volume, matrix metalloproteinase-9, tissue inhibitor of metalloproteinase-1

## Abstract

This study investigated the diagnostic accuracy of plasma biomarkers—specifically, matrix metalloproteinase (MMP-9), tissue inhibitor of metalloproteinase (TIMP-1), CD147, and the MMP-/TIMP-1 ratio in patients with Alzheimer’s disease (AD) dementia. The research cohort comprised patients diagnosed with probable AD dementia and a control group of cognitively unimpaired (CU) individuals. Neuroradiological assessments included brain magnetic resonance imaging (MRI) following dementia protocols, with subsequent volumetric analysis. Additionally, cerebrospinal fluid (CSF) AD biomarkers were classified using the A/T/N system, and apolipoprotein E (*APOE*) ε4 carrier status was determined. Findings revealed elevated plasma levels of MMP-9 and TIMP-1 in AD dementia patients compared to CU individuals. Receiver operating characteristic (ROC) curve analysis demonstrated significant differences in the areas under the curve (AUC) for MMP-9 (*p* < 0.001) and TIMP-1 (*p* < 0.001). Notably, plasma TIMP-1 levels were significantly lower in *APOE* ε4+ patients than in *APOE* ε4− patients (*p* = 0.041). Furthermore, *APOE* ε4+ patients exhibited reduced hippocampal volume, particularly in total, right, and left hippocampal measurements. TIMP-1 levels exhibited a positive correlation, while the MMP-9/TIMP-1 ratio showed a negative correlation with hippocampal volume parameters. This study sheds light on the potential use of TIMP-1 as a diagnostic marker and its association with hippocampal changes in AD.

## 1. Introduction

Alzheimer’s disease (AD) stands as a principal cause of dementia, characterized by a spectrum of cognitive impairments and behavioral alterations that disrupt daily activities. Representing 60–70% of dementia cases, AD significantly contributes to the global burden of disability among individuals over 60 years of age [1]. In Serbia, the incidence of dementia was estimated at 130,000 cases in 2019, with projections indicating a rise to approximately 180,000 by 2050 [2]. AD pathogenesis is influenced by three primary factors: proteinopathy, neurodegeneration, and neuroinflammation. Central to the disease’s pathohistology is the accumulation of amyloid-beta (Aβ) in extracellular spaces and the formation of neurofibrillary tangles comprising hyperphosphorylated and misfolded tau proteins within nerve cell axons. These pathological processes result in progressive neuronal loss, brain atrophy, reduction in hippocampal volumes, and subsequent cognitive decline. AD predominantly occurs in two variants: a less common familial form with an early onset, and the sporadic form appearing later in life—late-onset AD (LOAD), frequently associated with the apolipoprotein E (*APOE*) gene variations [3]. Diagnostic methodologies for AD encompass both neuroimaging techniques, such as magnetic resonance imaging (MRI), and the analysis of cerebrospinal fluid (CSF) biomarkers, following the National Institute on Aging-Alzheimer’s Association (NIA-AA) guidelines [4]. The A/T/N classification system further refines the diagnostic process [5]. The clinical presentation of AD is categorized into three phases: the asymptomatic phase; followed by the stage of mild cognitive impairment (MCI); and lastly, dementia, characterized by a decline in activities of daily living (ADL) due to cognitive impairment [6,7,8].

Emerging studies highlight the pivotal role of neuroinflammation in AD pathogenesis, where microglial activation and subsequent cytokine release, including tumor necrosis factor (TNF-α) and interleukins, contribute to disease progression [9]. Moreover, numerous inflammation-related biomarkers are gaining attention as potential novel biomarkers associated with AD [10]. Matrix metalloproteinases (MMPs), which are zinc-dependent endopeptidases, are integral to the extracellular matrix (ECM) homeostasis. Microglia-secreted MMPs have the ability to catalyze Aβ and exacerbate brain inflammation [11]. MMP-9, a gelatinase group member, has garnered significant research interest due to its involvement in AD’s pathogenesis and progression. Primarily synthesized as pro-MMP-9, it undergoes activation by CD147 in the extracellular space. Tissue inhibitor of metalloproteinase (TIMP-1) is co-secreted and forms complexes with MMP-9, thereby playing a crucial role in inhibiting and regulating MMP-9 activity [12]. MMP-9’s interplay in Aβ clearance, blood–brain barrier (BBB) integrity, and tau pathology underscores its role in AD’s pathogenesis and progression [11,13].

The search for reliable biomarkers for AD is a critical area of research, especially considering the variability in biomarker levels. The inconsistency in circulating levels of MMP-9 and TIMP-1 in AD patients suggests that while these biomarkers hold potential, they may not yet provide the consistency needed for accurate diagnosis across diverse patient populations. Minimally invasive methods for identifying AD pathology are highly sought after, as they would allow for easier and potentially earlier diagnosis.

The *APOE* ε4 allele is known to be a significant genetic risk factor for AD, and its presence can influence the levels of various inflammation-related biomarkers [3,14,15]. Research into the interrelation between *APOE* ε4 carrier status, plasma biomarkers, and hippocampal volume is crucial, as hippocampal atrophy is a well-established indicator of AD. Volumetric measures of the hippocampus can provide insights into the extent of neurodegeneration and are used alongside other biomarkers to improve the accuracy of AD diagnosis [16,17,18]. Despite the recognized significance of *APOE* genotyping in assessing AD risk and patient stratification into *APOE* ε4+ and *APOE* ε4−, the intricate relationship involving *APOE* ε4 positivity, MMP-9, and TIMP-1 remains incompletely understood. Furthermore, the mechanisms by which MMP-9 and TIMP-1 influence neurodegeneration, the reduction in hippocampal volume, and cognitive decline are not yet fully elucidated. For a comprehensive understanding of these relationships, this study aimed to assess the diagnostic accuracy of plasma MMP-9, TIMP-1, CD147, and MMP-9/TIMP-1 ratio, as well as to analyze the interrelation between *APOE* ε4 carrier status, plasma biomarkers, and hippocampal volumetric measures in patients with AD dementia.

## 2. Results

### 2.1. Demographic, Clinical, Neuroimaging, and CSF Laboratory Parameters

Table 1 presents an overview of the demographic, clinical, neuroimaging, and CSF laboratory parameters for the study participants. Notably, there were no significant variations in gender distribution observed between patients and cognitively unimpaired (CU) individuals (χ2 = 0.007, *p* = 0.934). However, significant differences were observed in age (*p* < 0.001), *APOE* ε4 positivity (χ2 = 5.095, *p* = 0.024), and Mini-Mental State Examination (MMSE) scores (*p* < 0.001) between these two groups. There were no differences in age between *APOE* ε4+ and *APOE* ε4− patients (*p* = 0.686).

### 2.2. Plasma Levels of MMP-9, TIMP-1, CD147, and MMP-9/TIMP-1 Ratio in the Studied Population

**Figure 1:**
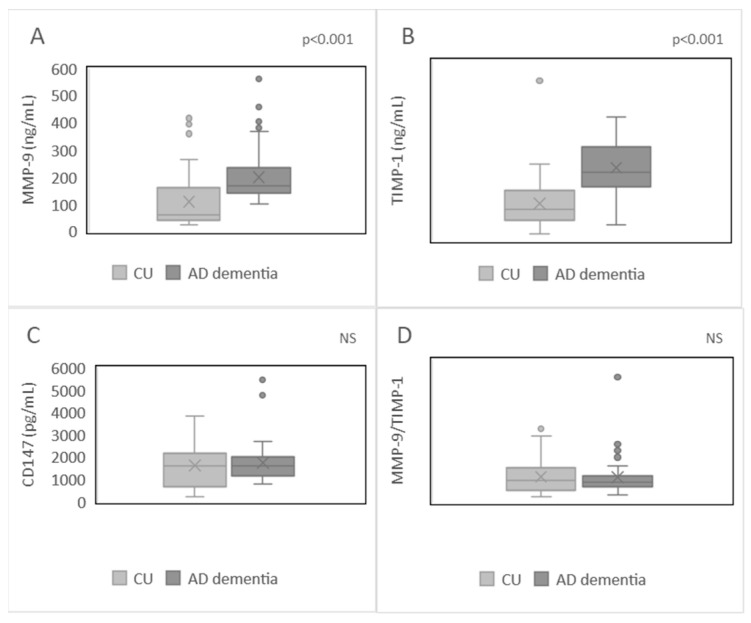
Plasma levels of MMP-9 (**A**), TIMP-1 (**B**), CD147 (**C**), and MMP-9/TIMP-1 ratio (**D**) in the patients and cognitively unimpaired (CU) individuals.

The study results indicate that patients with AD dementia exhibited elevated MMP-9 (*p* < 0.001, Figure 1A) and TIMP-1 levels (*p* < 0.001, Figure 1B) compared to CU individuals. However, no significant difference was observed in the level of CD147 or the MMP-9/TIMP-1 index between patients and controls (*p* = 0.560, Figure 1C; *p* = 0.958, Figure 1D, respectively). In the patient group, there was no observed correlation between MMP-9, TIMP-1, or the MMP-9/TIMP-1 ratio and Ab42/40 ratio (*p* = 0.255, *p* = 0.662, *p* = 0.514, respectively), p-tau levels (*p* = 0.815, *p* = 0.371, *p* = 0.857, respectively), or t-tau levels (*p* = 0.128, *p* = 0.904, *p* = 0.348, respectively).

### 2.3. Receiver Operating Characteristic (ROC) Curve Analysis

**Figure 2:**
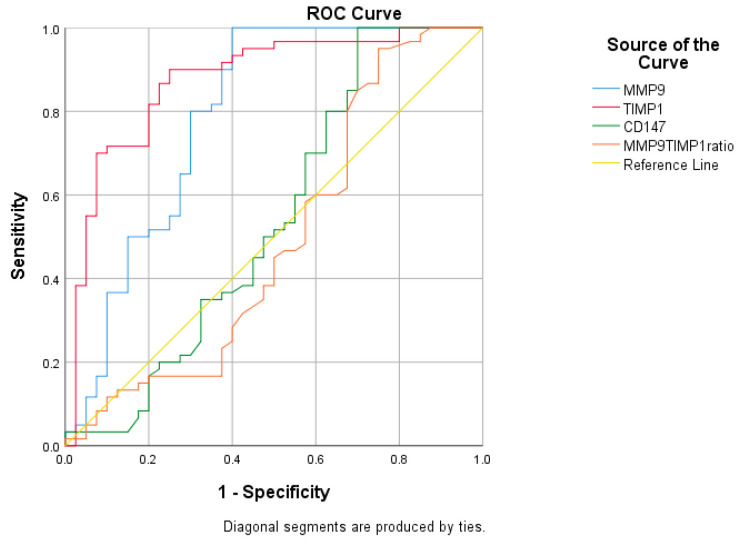
The receiver operating characteristic (ROC) curve analysis for plasma biomarkers.

The receiver operating characteristic (ROC) curve analysis indicated significant differences in the areas under the curve (AUC) for MMP-9 and TIMP-1 compared to the reference area (AUC = 0.791, *p* < 0.001; AUC = 0.875, *p* < 0.001, respectively). However, there were no significant differences in the areas under the curve for CD147 and the MMP-9/TIMP-1 ratio (AUC = 0.541, *p* = 0.490; AUC = 0.491, *p* = 0.877, respectively; Figure 2).

ROC curve analyses revealed that the cut-off value for MMP-9 was 92.14 ng/mL, and for TIMP-1 it was 233.80 ng/mL. These values effectively distinguished between AD dementia patients and controls. The sensitivity was 100% for MMP-9 and 90% for TIMP-1, while specificity stood at 60% for MMP-9 and 75% for TIMP-1. The Youden index was 0.60 for MMP-9 and 0.65 for TIMP-1. Additionally, when comparing the AUC for different plasma biomarkers, statistically significant differences were observed. Specifically, AUC differed significantly between MMP-9 and CD147 (z = 3.463, *p* = 0.0005), MMP-9 and the MMP-9/TIMP-1 ratio (z = 2.811, *p* = 0.0049), TIMP-1 and CD147 (z = 5.155, *p* < 0.0001), and TIMP-1 and the MMP-9/TIMP-1 ratio (z = 5.455, *p* < 0.0001). However, there were no significant differences in AUC between MMP-9 and TIMP-1 (*p* = 0.09), or between CD147 and the MMP-9/TIMP-1 index (*p* = 0.6995) (Table 2).

### 2.4. Hippocampal Volumes and Plasma Levels of MMP-9, TIMP-1, CD147, and MMP-9/TIMP-1 Ratio in APOE-ε4-Positive and APOE-ε4-Negative Patients

**Figure 3:**
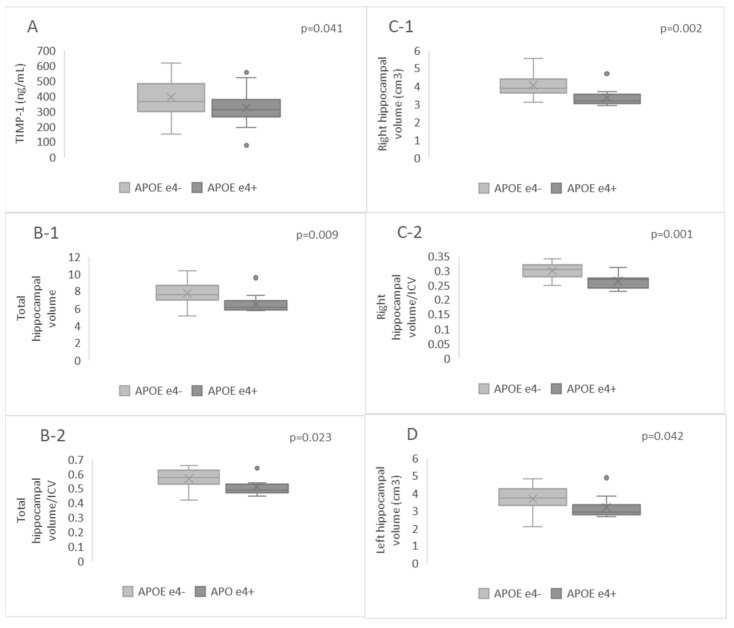
Plasma levels of TIMP-1 (**A**), and hippocampal volumes, including total hippocampal volume (**B-1**), total hippocampal volume/ICV (**B-2**), right hippocampal volume (**C-1**), right hippocampal volume/ICV (**C-2**), and left hippocampal volume (**D**) in *APOE* ε4+ and *APOE* ε4− patients.

Plasma TIMP-1 levels exhibited a significant decrease in *APOE* ε4+ patients when compared to *APOE* ε4− patients (*p* = 0.041, Figure 3A). There was no significant difference in MMP-9 or CD147 levels, nor in the MMP-9/TIMP-1 index, between patients who were carriers of the ε4 allele and non-carriers (*p* = 0.667, *p* = 0.961, *p* = 0.256, respectively).

A volumetric analysis of the hippocampus revealed that *APOE* ε4+ patients had significantly decreased hippocampal volume. Specifically, this decrease was observed in total hippocampal volume (*p* = 0.009, Figure 3B-1), total/intracranial cavity volume (ICV, *p* = 0.023, Figure 3B-2), right hippocampal volume (*p* = 0.002), Figure 3C-1), right hippocampal volume/ICV (*p* = 0.001, Figure 3C-2), and left hippocampal volume (*p* = 0.042, Figure 3D) when compared to *APOE* ε4− patients. However, there was no significant difference in the index left hippocampal volume/ICV (*p* = 0.159) between these two patient groups. We observed no differences in left hippocampal volume/ICV between males and females among *APOE* ε4+ patients (*p* = 0.321).

### 2.5. Correlation Analysis between Plasma Biomarkers and Hippocampal Parameters in AD Dementia Patients

**Figure 4:**
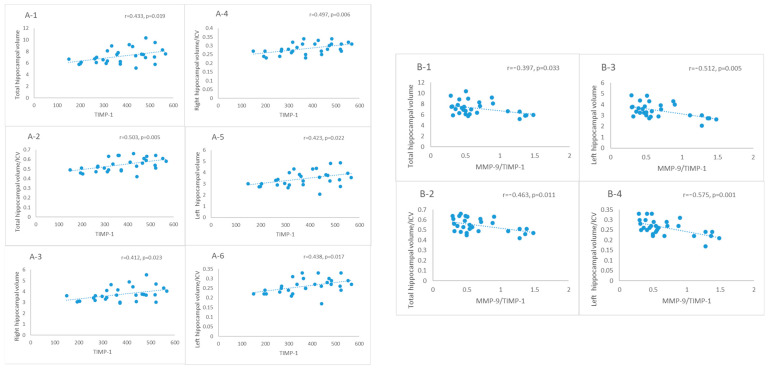
Correlation analysis between TIMP-1 and total hippocampal volume (**A-1**), total hippocampal volume/ICV (**A-2**), right hippocampal volume (**A-3**), right hippocampal volume/ICV (**A-4**), left hippocampal volume (**A-5**), and left hippocampal volume/ICV (**A-6**); MMP-9/TIMP-1 ratio and total hippocampal volume (**B-1**), total hippocampal volume/ICV (**B-2**), left hippocampal volume (**B-3**) and left hippocampal volume/ICV (**B-4**) in AD dementia patients.

TIMP-1 levels demonstrated a positive correlation with several parameters: total hippocampal volume (r = 0.433, *p* = 0.019, Figure 4A-1), total hippocampal volume normalized to ICV (r = 0.503, *p* = 0.005, Figure 4A-2), right hippocampal volume (r = 0.412, *p* = 0.023, Figure 4A-3), right hippocampal volume normalized to ICV (r = 0.497, *p* = 0.006, Figure 4A-4), left hippocampal volume (r = 0.423, *p* = 0.022, Figure 4A-5), and left hippocampal volume normalized to ICV (r = 0.438, *p* = 0.017, Figure 4A-6).

Moreover, the MMP-9/TIMP-1 index exhibited a negative correlation with total hippocampal volume (r = −0.397, *p* = 0.033, Figure 4B-1), total hippocampal volume normalized to ICV (r = −0.463, *p* = 0.011, Figure 4B-2), left hippocampal volume (r = −0.512, *p* = 0.005, Figure 4B-3), and left hippocampal volume normalized to ICV (r = −0.575, *p* = 0.001, Figure 4B-4).

## 3. Discussion

The exploration of inflammation-related biomarkers in AD has revealed that certain biomarkers present in the plasma or CSF of AD patients may serve as promising indicators for both preclinical and clinical stages of the disease. Among these, MMP-9 and TIMP-1 are gaining recognition for their roles in maintaining BBB integrity, modulating neuroinflammation, and facilitating the clearance of Aβ peptides [10]. MMP-9 is pivotal in the breakdown and removal of Aβ, with heightened expression noted in the brains of AD patients. Previous research has demonstrated MMP-9’s ability to target tau protein and induce tau pathology. Furthermore, this proteinase impacts endothelial tight junction proteins, alters pericyte phenotypes, and elevates the permeability of the BBB [11]. Nevertheless, the variations in plasma MMP-9 and TIMP-1 levels among AD patients have yielded inconsistent findings. This study’s results suggest that individuals with AD dementia demonstrated increased MMP-9 and TIMP-1 plasma levels in comparison to cognitively unimpaired individuals. The findings of this study are consistent with those of Lorenzl et al. [19,20], which reported significantly increased plasma levels of MMP-9 in AD patients in comparison to both controls and individuals with MCI-AD. In contrast, conflicting findings have been reported in other studies, suggesting that plasma MMP-9 levels in AD patients showed a significant decrease compared to healthy subjects [21]. Additionally, some investigations have not identified a significant difference in MMP-9 levels or the MMP-9/TIMP-1 index between AD patients and cognitively unimpaired subjects [10,22]. In a recent study by Liu et al. [23], plasma MMP-9 levels and the MMP-9/TIMP-1 ratio exhibited a significant increase in AD patients compared to healthy controls. In our study, ROC curve analysis indicated that MMP-9 and TIMP-1 displayed moderate diagnostic accuracy in discerning dementia due to AD from CU subjects, according to the Swets criterion [24]. The findings from Liu et al. [23] demonstrate that the MMP-9/TIMP-1 ratio exhibited superior diagnostic performance compared to MMP-9 alone in identifying AD. The AUC for the MMP-9/TIMP-1 ratio was 0.906, indicating high diagnostic accuracy, with sensitivity and specificity at 95.8% and 75%, respectively. Our research results align with previous studies demonstrating elevated levels of pro-inflammatory cytokines in AD patients’ CSF [9,10,25], with consequent MMP-9 synthesis, while increased TIMP-1 levels could signify a compensatory mechanism aimed to counterbalancing heightened MMP-9 production. The disruption observed in these plasma biomarkers may mirror underlying cerebral pathological mechanisms, thereby presenting valuable insights into AD diagnosis and disease progression. On the other hand, AD dementia patients in this study were significantly older than CU individuals, and increased MMP-9 levels could be a consequence of age differences, which could be considered a limitation of the study. However, while some studies have reported increased MMP-9 levels in older individuals and AD patients compared to younger and cognitively healthy individuals, the direct influence of age on MMP-9 production in AD remains a subject of ongoing research and debate.

APOE is a glycoprotein synthesized by the *APOE* gene and is pivotal in Aβ clearance from the brain via BBB transport, enzymatic degradation, and cellular mechanisms, including cleavage by lipoprotein receptors [26]. The APOE4 isoform demonstrates diminished Aβ affinity, hindering clearance mechanisms and leading to Aβ accumulation and disease progression [3,27]. Furthermore, *APOE* ε4 carrier status correlates with tau, α-synuclein, and other neurodegenerative protein deposits, independent of Aβ [3,26,28,29]. Moreover, the presence of the *APOE* ε4 allele has been linked to a heightened susceptibility to AD in comparison to the reference allele *APOE* ε3 [3,30], as demonstrated in our previous study [14].

The APOE ε4 allele has been linked to cognitive and pathological heterogeneity in AD. It is underscored that *APOE* ε4 not only impacts susceptibility to AD but also influences differences in clinical presentation. *APOE* ε4+ AD patients tend to exhibit more pronounced memory impairment. In contrast, *APOE* ε4− AD patients demonstrate greater deficits in language, executive function, and spatial cognition [3,31].

Atrophy serves as a sensitive marker of neurodegeneration, and previous findings indicate that individuals carrying the *APOE* ε4+ allele experience volume reductions in a structure crucial for memory formation—the hippocampus. It is suggested that the hippocampus can be susceptible to neurodegeneration as *APOE* ε4 carriers progress into middle and old age. While reductions in hippocampal volume were observed bilaterally, the atrophy was particularly conspicuous in the right hippocampus. Specifically, there was a notable decrease in volume detected in the right hippocampus of *APOE* ε4 carriers, whereas a borderline reduction in volume was observed in the left hippocampus of *APOE* ε4− individuals [32]. Additionally, it was shown that advancing age and the presence of the *APOE* ε4 allele are linked to accelerated rates of hippocampal volume reduction and episodic memory decline [33]. Our study findings align with these observations, demonstrating significant reductions in nearly all hippocampal volume measurements among *APOE* ε4+ patients compared to *APOE* ε4− patients, except for the index, left hippocampal volume/ICV.

In this study, plasma TIMP-1 levels were lower in *APOE* ε4+ AD dementia patients compared to *APOE* ε4− patients. Furthermore, TIMP-1 levels showed positive correlations with all analyzed parameters in hippocampal volumetry. In contrast, the MMP-9/TIMP-1 ratio demonstrated negative correlations with both total and left hippocampal volume, whether in absolute measurements or when normalized to ICV.

It was shown that inflammatory biomarkers, such as plasma glial fibrillary acidic protein (GFAP), were linked to hippocampal volume loss in elderly CU individuals [34]. Furthermore, elevated MMP-9 activity was observed in the brains of patients with AD and MCI along with a negative correlation between MMP-9 levels and MMSE scores [35]. Additionally, increased levels of CSF MMP-9 were found in individuals exhibiting low Aβ levels, high tau levels, and *APOE* ε4 positivity, compared to those with negative biomarkers, indicating a significance of MMP-9 in the early pathophysiology of AD, even preceding cognitive decline [36]. Plasma MMP-9 and MMP/9/TIMP-1 ratio in AD patients showed a positive correlation with p-tau and a negative correlation with Aβ42/Aβ40 ratio [23]. Moreover, a reduction in the TIMP-1/MMP-9 ratio among AD patients correlated with elevated t-tau levels, indicative of neurodegeneration [36]. However, we did not find a correlation between MMP-9, TIMP-1, or the MMP-9/TIMP-1 ratio and t-tau levels in our study. This lack of correlation could be attributed to factors such as sample size, fluctuations in biomarker levels, and the stage of the disease at which the biomarkers were analyzed. In a study investigating hippocampal volumes and cognitive function over a four-year follow-up period utilizing brain MRI scans and MMSE assessments, individuals with MCI-AD, especially those with elevated baseline levels of MMP-9, demonstrated accelerated declines in hippocampal volumes in comparison to patients with moderate and low MMP-9 levels [10]. Moreover, these findings were particularly pronounced in *APOE* ε4+ patients [37]. These data indicate MMP-9 as a promising biomarker of neurodegeneration in AD and a potential treatment target for this disease.

In our previous research, the *APOE* ε4 allele has been linked to increased levels of TNF-α [15] and IL-1β [38], as well as compromised BBB integrity [39] and dysfunction of tight junctions [40]. Pericytes and MMP-9 have been proposed as potential mediators of these effects [26]. Emerging evidence suggests that APOE may modulate brain MMP-9 activity, contributing to increased MMP-9 levels in response to APOE4 isoform [41]. Furthermore, Shackleton et al. [42] emphasized the influence of the dosage-dependent MMP-9 treatment on heightened lipoprotein receptor proteolysis in APOE4 transgenic mice, resulting in increased beta-amyloid elimination through the BBB when MMP-9 inhibitor was present.

In contrast, treatment with TIMP-1 resulted in decreased Aβ accumulation in both the hippocampus and cerebral cortex in the AD model. TIMP-1 was shown to improve Aβ-induced cognitive decline by restoring the PI3K/Akt signaling pathway and maintaining synaptic function [43]. Several studies have reported elevated levels of TIMP-1 in the CSF of patients with AD and Parkinson’s disease (PD), indicating a protective effect of TIMP-1 [23,44]. In line with these results, the positive correlation observed between TIMP-1 and hippocampal volumes, along with the negative correlation between the MMP-9/TIMP-1 ratio and hippocampal volumes in our study, underscores the potential protective role of TIMP-1 in neurodegeneration and disease progression. Notably, the *APOE* ε4 allele may mediate and accelerate these processes. However, the exact mechanisms driving this process remain unclear.

The regulation of MMP activity by TIMPs suggests that dysregulation of TIMPs may contribute to the progression of AD [45]. Furthermore, variations in the TIMP-1 gene have been established to influence the risk of neurodegenerative diseases and gene expression [46]. The presence of the *TIMP-1* rs4898 C allele has been associated with protective effects in PD [47]. In addition, CCL5 is a protein that interacts with G protein-coupled receptors on both astrocytes and neurons. Studies have indicated that APOE4 is associated with increased CCL5 production and CCL5/CCR5 signaling, along with elevated TIMP-1 levels in astrocytes, resulting in a reduction in MMP-9 effects. Moreover, decreasing CCR5 expression might decrease astrocytic TIMP-1 levels, thereby enabling MMP-9 to enhance its effects [46]. Given that biochemical, neurophysiological, and behavioral deficits in heterozygous APOE4 TR mice are reversed by heterozygous knockout of CCR5, it is conceivable that genetic variations in the CCR5 gene could influence TIMP-1 expression.

Our findings support the role of MMP-9 in hastening the receptor proteolysis essential for the clearance of Aβ (LDLR and LRP1), induction of tau pathology, and neurodegeneration [42,48]. The decreased expression of TIMP-1 and diminished inhibition of MMP-9 in *APOE* ε4+ patients in our study could be a consequence of the presence of genetic variations in the TIMP-1 gene and other genes associated with neuroinflammation (*CCR5*), suggesting a weakened neuroprotective effect of TIMP-1 in this patient group. Therefore, our findings not only offer insight into the functional role of TIMP-1, as an inhibitor or MMP-9, with potential neuroprotective effects, but also support its potential as a key molecule in cytokine-mediated treatment for AD. Furthermore, utilizing ultra-sensitive assay technologies is imperative for evaluating core biomarkers in plasma. Recent studies have underscored the diagnostic potential of plasma biomarkers such as the Aβ42/40 ratio, t-tau, p-tau181, p-tau217, neurofilament light (NfL), and GFAP in AD [49,50,51]. Nonetheless, further research is necessary to standardize the levels of these blood-based biomarkers across the general population and to contextualize our findings in relation to these biomarkers.

In limitations, due to ethical and medical constraints, we conducted lumbar puncture, brain MRI scans, and evaluation of hippocampal volumes exclusively in the patient group, and the differences in these parameters between patients and controls were unable to be observed. In addition, given the limited sample size, we refrained from analyzing the differences in biomarker levels between *APOE* ε4+ and *APOE* ε4− individuals among the CU subjects. This decision was made due to the small number of *APOE* ε4+ individuals in the CU group, which could compromise the statistical power of the analysis. Furthermore, the correlation analysis between MMP-9, TIMP-1, and the MMP-9/TIMP-1 ratio with Ab42/40, p-tau, and t-tau was not conducted in AD patients compared to CU individuals.

## 4. Materials and Methods

This study comprised 60 patients with a diagnosis of probable AD dementia and the control group, which consisted of 40 cognitively unimpaired (CU) individuals. The clinical evaluation of patients, as well as the inclusion and exclusion criteria, can be found in our previously published article [14]. Cognitively unimpaired individuals met the criteria established by neuropsychological test scores falling within the normative range relative to an individual’s age, sex, and educational background. Additionally, they obtained an MMSE score equal to or greater than 26.

### 4.1. Processing of CSF Samples and Subsequent Biomarker Assessments

CSF samples were collected from all patients using polypropylene tubes, followed by centrifugation at 2000× *g* for 10 min at +4 °C. The supernatant was then separated, aliquoted, and stored at −80 °C until analysis. Biomarkers in the CSF were analyzed using enzyme-linked immunosorbent assay (ELISA) kits from EUROIMMUN, Lubeck, Germany, following the manufacturer’s instructions. This included the assessment of the Aβ42/40 ratio, total tau (t-Tau), and phosphorylated tau (p-Tau), as we have previously described [14]. Biomarker positivity was assessed by the A/T/N classification [5].

### 4.2. Blood Sample Preparation, Genotyping, and Biomarker Analyses

From the whole blood with EDTA as an anticoagulant, the DNA was isolated. APOE genotyping was conducted via real-time PCR to determine the *APOE* ε4 allele carrier status, as we have previously described [14]. Patients carrying at least one *APOE* ε4 allele were assigned as *APOE* ε4 positive (*APOE* ε4+), while patients carrying *APOE* ε2 or ε3 alleles were considered *APOE* ε4 negative (*APOE* ε4−).

Blood samples were centrifuged at 2000 × *g* for 10 min at +4 °C. Plasma was separated and frozen at −80 °C. Levels of MMP-9, TIMP-1, and CD147 were determined in the plasma by ELISA according to the manufacturer’s instructions (R&D Systems, Abingdon, UK). The levels of MMP-9 and TIMP-1 were expressed in ng/mL, while the level of CD147 was expressed in pg/mL. After determining the levels of MMP-9 and TIMP-1, their ratio (MMP-9/TIMP-1) was also determined. The minimum detectable dose (MDD) for MMP-9 was 0.156 ng/mL, for TIMP-1 0.08 ng/mL, and for CD147 2.94 pg/mL.

### 4.3. Neuroradiological Examination

The neuroradiological examination started with a brain MRI conducted according to the protocol for dementia and volumetric study [18]. All the patients underwent examination at the Center for Radiology in University Clinical Center Nis on General Electronic Healthcare (GE) SIGNA PIONEER MRI scanner with a 3T field strength. The leading sequence used in the volumetric analysis was three-dimensional axial T1 weighted (3D T1w) BRAVO sequences. The acquisition parameters included TR: 8.6 ms; TE: 3.2 ms; center flip angle: 12°; number of excitations (NEX): 1; bandwidth: 31.25 kHz; voxel resolution: 1 × 1 × 1 mm; slice thickness: 1 mm; matrix: 250 × 250; scan time: 3′35 min.

### 4.4. Volumetric Analysis

We utilized VolBrain [52], an online MRI volumetry system for whole-brain segmentation. The volBrain platform is an entirely automatic pipeline for volume analyses founded on multi-atlas label fusion technology, which can provide information about brain volumes. This process is available via the volBrain online interface. We employed the Vol2Brain pipeline [53], which delivers the volumes of brain tissues, lobes, 135 different structures, and asymmetry indexes. The pipeline demands an anonymized and compressed 3D T1w MRI brain sequence in NIFTI format, so we converted the DICOM images for each scan. As the output, we obtained a package that included some image files and two (CSV and PDF) reports delivering all the volumetry values calculated from the segmentations. The reports contain several snapshots from the various labeling steps as quality control. The gender and years of the life of the submitted patients were provided, so we obtained average volumes and asymmetry reference values for every section of the brain for comparisons. These reference boundaries were automatedly estimated from the IXI dataset [18], covering almost all adult lifespans. Hippocampal volumes are delivered in absolute values (cm^3^) and relative ones with regard to the ICV.

#### Statistical Analysis

All continuous variables were expressed as mean ± SD. The differences between demographic and clinical parameters in the patients and CU individuals were evaluated by t-test and chi-squared (χ2) test, as appropriate. To identify the differences in continuous variables between *APOE*ε4+ and *APOE*ε4− patients, we used an independent *t*-test. Bivariate associations between continuous variables were examined using Pearson’s r correlation coefficient. The diagnostic accuracy of the biomarkers was examined using receiver-operating characteristic (ROC) curves created for each investigated biomarker. Areas under each curve (AUC) with 95% confidence intervals (95% CI) were calculated. Based on AUC values, the diagnostic accuracy of biomarkers was classified as low (0.50–0.70), moderate (0.71–0.90), or high (>0.90) [24]. The cut-off value with the best combination of sensitivity and specificity was determined for each biomarker using the Youden index, calculated by subtracting 1.0 from the sum of sensitivity and specificity [54]. A comparison of areas under various ROC curves was conducted by the DeLong method [55]. A value of *p* < 0.05 was considered statistically significant. Statistical analyses were performed using SPSS version 20.0 (SPSS Inc., Chicago, IL, USA). An a priori power analysis was performed using G*Power version 3.1.9.7 to ascertain the minimum sample size necessary to test the study hypothesis. The analysis revealed that a sample size of N = 20 per group (N = 40 in total) would provide 80% power to detect large effects (d = 0.9) based on a pilot study, with a significance criterion of α = 0.05, using a two-sample *t*-test. Therefore, the obtained sample size of N = 60 for the patient group and N = 40 for the control group (N = 100 in total) is sufficient to test the study hypothesis.

## 5. Conclusions

Our study highlights MMP-9 and TIMP-1 as potential biomarkers with moderate diagnostic accuracy for distinguishing dementia due to AD from cognitively unimpaired individuals. We also observed that patients with *APOE* ε4 positivity exhibited reduced hippocampal volume and decreased TIMP-1 levels. Notably, TIMP-1 levels showed a positive correlation with hippocampal volume, while the MMP-9/TIMP-1 ratio exhibited a negative correlation with total and left hippocampal volume measurements. These findings suggest that TIMP-1 may hold promise as a blood-based biomarker, potentially reflecting the protective role of this inhibitor in neurodegeneration among patients with AD dementia. Nevertheless, it is crucial to highlight that additional evidence is necessary to comprehensively grasp the exact roles of these biomarkers in disease progression, especially concerning neurodegeneration mechanisms. This involves exploring their correlation with CSF and plasma NfL, plasma p-tau181, p-tau217, and GFAP levels, using ultrasensitive detection technologies, examining their relationship with volumetric analyses of substructures of other brain regions to evaluate atrophy as a marker of neurodegeneration, as well as investigating genetic variations that could influence TIMP-1 expression in AD. Further research is needed to elucidate these aspects and enhance our understanding of AD pathology.

## Figures and Tables

**Table 1:** Demographic, clinical, neuroimaging, and CSF laboratory parameters of the study participants.

Parameters	CU	AD Dementia
Sex (Female, N (%))	23 (57.5)	35 (58.33) #
Age (years, M (SD))	59 (4.15)	70.80 (7.66) *
MMSE (M (SD))	28.72 (1.01)	16.51 (6.87) *
Hippocampal volumes		
*Total (cm^3^, M (SD))*	-	7.19 (1.28)
*Total/ICV (M (SD))*	-	0.54 (0.06)
*Right (cm^3^, (M (SD))*	-	3.74 (0.64)
*Right/ICV (M (SD))*	-	0.28 (0.03)
*Left (cm^3^, (M (SD))*	-	3.45 (0.67)
*Left/ICV (M (SD))*	-	0.26 (0.03)
APOE status		
*APOE ε4 positive (N (%))*	8 (20)	25 (41.66) ** #
CSF biomarkers		
*Aβ42/Aβ40 ratio (M (SD))*	-	0.07 (0.01)
*t-Tau (pg/mL, M (SD))*	-	703.89 (254.86)
*p-Tau 181 (pg/mL, M (SD))*	-	162.47 (69.47)

AD—Alzheimer’s disease, CSF—cerebrospinal fluid, CU—cognitively unimpaired, ICV—intracranial cavity volume, MMSE—Mini-Mental State Examination; * *p* < 0.001, ** *p* = 0.024 vs. CU; # χ2 test was used.

**Table 2:** Sensitivity and specificity of plasma biomarkers.

Biomarker	AUC	95% CI (LB-UB)	*p*-Value	Cut-Off	Sensitivity (%)	Specificity (%)	Difference between AUC (*p*-Value)
MMP-9 (ng/mL)	0.791	0.690–0.892	<0.0001		100	60	0.09 vs. TIMP-1
				92.14			0.0005 vs. CD147
							0.0049 vs. MMP-9/TIMP-1
TIMP-1 (ng/mL)	0.875	0.801–0.949	<0.0001	233.80	90	75	<0.0001 vs. CD147
							<0.0001 vs. MMP-9/TIMP-1
CD147 (pg/mL)	0.541	0.415–0.667	0.490	/	/	/	0.6995 vs. MMP-9/TIMP-1
MMP-9/TIMP ratio	0.491	0.366–0.615	0.877	/	/	/	/

AUC—area under the curve, CI—confidence interval, LB—lower bound, UB—upper bound, MMP-9—matrix metalloproteinase-9, TIMP-1—tissue inhibitor of metalloproteinase-1.

## Data Availability

The data used to support the findings of this study are included in the article; further inquiries can be directed to the corresponding author.
